# Incorporating seascape connectivity in conservation prioritisation

**DOI:** 10.1371/journal.pone.0182396

**Published:** 2017-07-28

**Authors:** Rebecca Weeks

**Affiliations:** Australian Research Council Centre of Excellence for Coral Reef Studies, James Cook University, Townsville, Queensland, Australia; University of Sydney, AUSTRALIA

## Abstract

In conservation prioritisation, it is often implicit that representation targets for individual habitat types act as surrogates for the species that inhabit them. Yet for many commercially and ecologically important coral reef fish species, connectivity among different habitats in a seascape may be more important than any single habitat alone. Approaches to conservation prioritisation that consider seascape connectivity are thus warranted. I demonstrate an approach that can be implemented within a relatively data-poor context, using widely available conservation planning software. Based on clearly stated assumptions regarding species’ habitat usage and movement ability, this approach can be adapted to different focal species and contexts, or refined as further data become available. I first derive a seascape connectivity metric based on area-weighted proximity between juvenile and adult habitat patches, and then apply this during spatial prioritisation using the decision-support software Marxan. Using a case study from Micronesia, I present two applications: first, to inform prioritisation for a network of marine protected areas to achieve regional objectives for habitat representation; and second, to identify nursery habitat patches that are most likely to supply juveniles to adult populations on reefs within existing protected areas. Incorporating seascape connectivity in conservation prioritisation highlights areas where small marine protected areas placed on coral reefs might benefit from proximity to other habitats in the seascape, and thus be more effective. Within the context of community tenure over resources, identification of critical nursery habitats to improve the effectiveness of existing marine protected areas indicates where collaboration across community boundaries might be required. Outputs from these analyses are likely to be most useful in regions where management is highly decentralised, imposing spatial constraints on the size of individual protected areas.

## Introduction

There has recently been a perceptual shift away from habitat representation as the sole or primary focus of conservation prioritisation, towards consideration of ecological processes that shape the distribution and abundance of biodiversity features [1–6]. In marine ecosystems, connectivity processes are paramount [7], and designing systems of marine protected areas that maintain connectivity between habitat patches has long been considered an objective of conservation planning [1,8]. Two forms of connectivity are critical to structuring coral reef fish populations [9]: dispersal of larvae in the pelagic environment [10], and post-settlement migration by individuals across the seascape [11]. Whilst a growing literature has described approaches for considering larval connectivity in conservation prioritisation [e.g. 12–15], relatively less attention has been directed towards developing and applying methods for considering post-settlement connectivity [16,17].

Seascape connectivity (connectedness among different habitats in a seascape, *c*.*f*. among patches of the same habitat type [18]) is essential for species that utilise more than one habitat, either during diurnal movements or at different stages in their life history. Mangroves, seagrass beds, and lagoon reefs provide nursery areas for many commercially and ecologically important fish species that subsequently make ontogenetic shifts to adult populations on coral reefs [19–22]. These ‘back-reef’ habitats are often overlooked for conservation or management in favour of coral reefs that support greater adult biomass, yet they can be equally if not more at risk from habitat degradation and loss [23–25]. Even where juveniles are not targeted by fishers, they can be vulnerable to habitat degradation, for example from sedimentation caused by poor land-use practices [26].

There is clear empirical evidence that proximity to nursery habitats can enhance the effectiveness (i.e. increasing the abundance, density, or biomass of fish species) of marine protected areas on coral reefs [18,27–30]. For example, at study sites across the western Pacific, the abundance of harvested fish species was significantly greater on protected reefs close to mangroves, but not on protected reefs isolated from mangroves [29]. The functional role of herbivorous fish species that perform ontogenetic migrations may also enhance the resilience of coral reefs close to mangroves [31,32]. Despite this evidence, and widespread calls to account for connectivity among habitats in the design of spatial management (e.g. [21,29,30]), there remain few examples where seascape connectivity is explicitly considered in spatial conservation prioritisation (the analytical process of identifying priority areas for conservation or management actions).

Possible reasons for this include a lack of empirical data and poor mechanistic understanding of the nature of ontogenetic migration and nursery habitat function [20]. Whilst frameworks for better understanding seascape connectivity have been proposed (e.g. [20,25]), these remain aspirational in many conservation contexts due to their data and/or resource requirements. In contrast, in terrestrial systems landscape ecology has long been considered in conservation prioritisation [33], and software facilitates the identification of protected area networks that account for structural connectivity via habitat corridors [34].

Rules of thumb for protecting species that undergo ontogenetic habitat shifts advise protecting some portion of each habitat used throughout ontogeny, ideally within a single large marine protected area, and where this is not possible, within multiple small protected areas that are spaced to allow for movement among habitats [35]. Such guidelines have typically been operationalised through objectives to achieve threshold levels of representation for individual habitat types (e.g. [36]). In contrast, a seascape ecology approach would consider the functionality provided by mosaics of different habitat types [37].

Theoretical literature on marine protected area design has largely ignored seascape connectivity, modelling fish populations with a pelagic larval stage and a relatively sedentary adult stage e.g. [38,39]. A small number of modelling studies have investigated the efficacy of marine protected areas for ontogenetically migrating species, under different assumptions about fish movement, density dependence, and spatial patterns of exploitation and management (e.g. [16,40]). However, resulting insights point to remaining empirical uncertainties, and have yet to be incorporated into planning frameworks.

A few studies have come closer to explicitly considering seascape connectivity in conservation planning. Mumby [24] proposed several algorithms for quantifying connectivity between coral reefs and mangrove habitats at the seascape scale. Building upon this work, Edwards *et al*. [41] considered connectivity between reefs and mangroves in conservation prioritisation by adjusting the expected biomass of fish species on reefs proximate to mangroves, and including this value in a modified objective function within Marxan’s reserve-selection algorithm. More recently, Engelhard *et al*. [42] used a network analytic approach to quantify connectivity among habitat mosaics (at the scale of home range movements rather than ontogenetic migrations) and applied the resulting metrics to evaluate the effectiveness of an existing system of protected areas.

Here, I demonstrate an approach to incorporating seascape connectivity in conservation prioritisation that can be implemented using widely available conservation planning software and within a relatively data-poor context. Based on a conceptual model of seascape connectivity, this approach can easily be refined if and when data become available to validate (or refute) explicitly stated assumptions. The aim is to prioritise for protection areas that will facilitate the supply of juveniles from protected nursery habitats to protected areas on adult reef habitats, increasing the efficacy of spatial management in regions where protected areas cannot reasonably encompass the extent of species’ ontogenetic movements. This focal species approach might be applied either to inform the design of new marine protected areas, or the adaptive management of existing marine protected areas. I illustrate the approach through application to a case study region in Micronesia.

## Methods

To prioritise for protection nursery and adult habitat patches that are connected within the spatial extent of species’ movement capabilities I first derive a seascape connectivity metric, based on area-weighted proximity between habitat patches, and then apply this during spatial prioritisation using the decision-support software Marxan [43]. The seascape connectivity metric combines physical attributes of the seascape (i.e. the spatial configuration of habitat patches) with information on the movement ability (estimates of ontogenetic migration distance) of a focal coral reef fish species, and is thus considered a metric of “potential connectivity” *sensu* Calabrese & Fagan [44]. To facilitate incorporation in spatial prioritisation, the seascape connectivity metric is calculated for planning units, which form the units of selection during prioritisation.

I present two applications of the seascape connectivity metric: first, to inform prioritisation for a network of marine protected areas to achieve regional objectives for habitat representation; and second, to identify critical nursery habitats to improve the effectiveness of existing marine protected areas within the context of adaptive management.

### Case study

**Fig 1:**
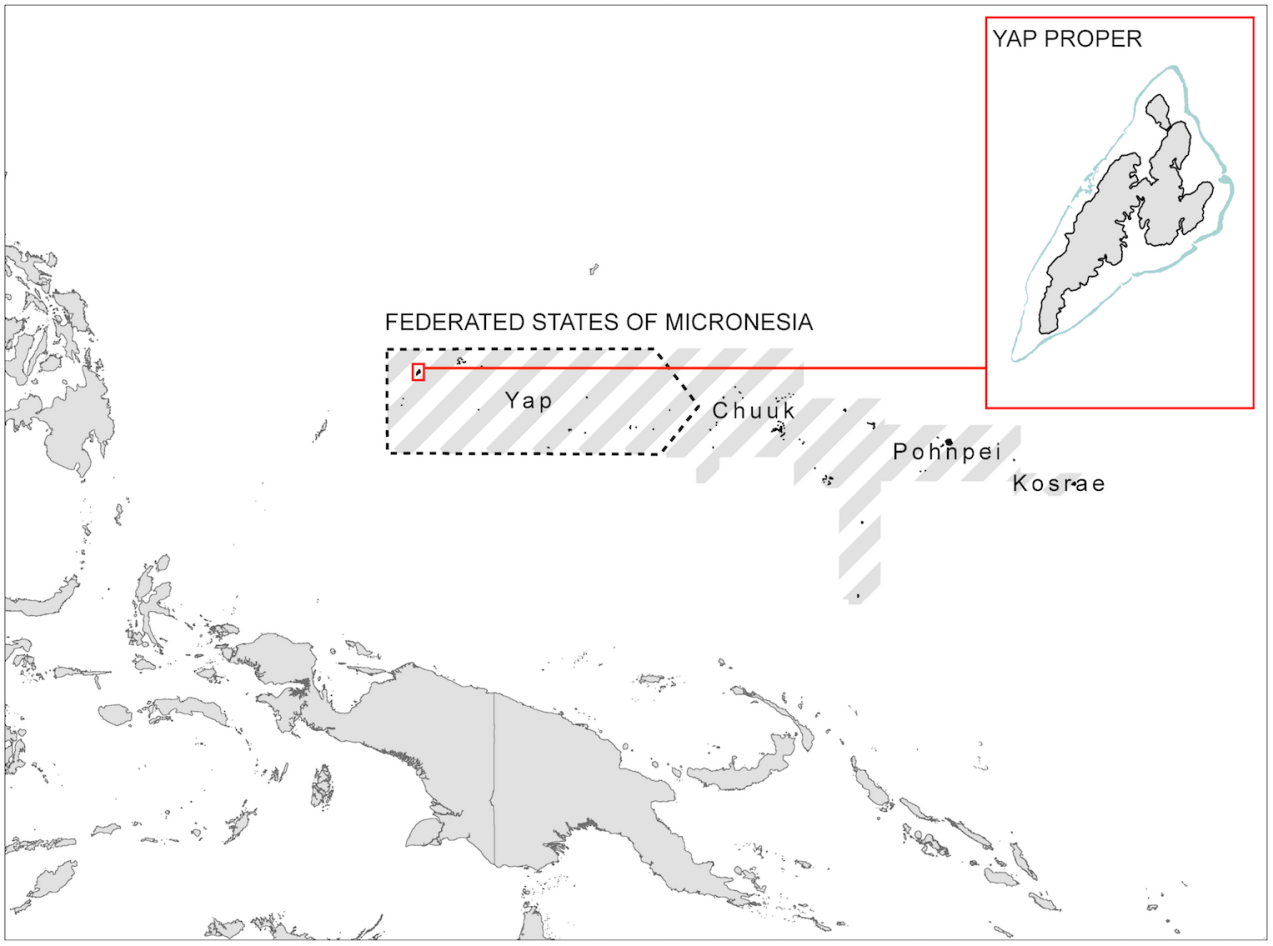
The location of the case study region, Yap Proper, within the Federated States of Micronesia.

Westernmost of the four constituent states of the Federated States of Micronesia, Yap consists of a cluster of four high islands connected by mangroves (sometimes referred to as ‘Yap proper’) and a number of low atolls and islets (collectively referred to as the ‘outer islands’), spread 1000 km to the east and south across the western Pacific Ocean (Fig 1). Yap proper (the focal region for the analyses) is surrounded by a single, continuous reef system, c.30 km long and up to 15 km across. The seascape exhibits a broad pattern of zonation: extensive seagrass meadows give way to a predominantly sandy zone with scattered algae and corals, which extends out to the barrier reef. Enclosed lagoons within the reef flat (locally known as blue holes) contain well-developed coral communities [45] and provide sheltered habitat for juvenile and adult reef fish [46].

Improved fishing technology, growing infrastructure on land, and increased reliance upon a cash economy represent threats that are not readily addressed by traditional management in place [45]. A desire exists to develop a state-wide protected area network for Yap. However, all natural resources are owned by communities [47], and thus the state has no jurisdiction to impose spatial management. Though there are a number of existing, well-managed marine conservation areas, community boundaries impose constraints on the spatial extent at which management can be implemented. Local communities are primarily interested in identifying how marine protected area placement and design can maximise local fisheries benefits. A seascape connectivity approach offers a way to address these concerns within a state-wide conservation prioritisation framework.

Several species of fisheries and cultural importance in Yap perform ontogenetic migrations between nursery and adult habitats. Notably, bumphead parrotfish (*Bolbometopon muricatum*) have high cultural significance in Micronesia, are targeted by local fishers [45], and are highly vulnerable to nursery habitat degradation [26]. Whilst the level of dependence upon nursery habitats remains uncertain for many species [20,48], there is good empirical support for ontogenetic habitat requirements in *B*. *muricatum* [49]. In Yap, bumphead parrotfish are found only on the reefs around Yap proper, and are absent from outer island reefs where mangroves are also absent. In Solomon Islands, Olds *et al*. [50] found that marine protected areas designed to conserve bumphead parrotfish enhanced the abundance of 17 other reef fish species, highlighting their suitability as a focal species for conservation planning. Other locally important fishery species in Yap, including the dusky rabbitfish, *Siganus fuscescens*, have been found to be more abundant on protected reefs near mangroves [27,28] and would thus likely benefit from a network of protected areas designed to maintain seascape connectivity.

### Seascape connectivity metric derivation

Widely available habitat maps (e.g. [51]) capture information on patch size, distribution, and configuration that can be used to determine the structural connectivity of a seascape. However, to be meaningful, a seascape connectivity metric must be ecologically informed [1,52] and scaled to the mobility of the species of interest [11]. Several assumptions are required, regarding: (1) the nature of nursery and adult habitat; (2) the maximum distance fish move between nursery and adult habitats; (3) migratory pathways taken between habitat patches; and the (4) effect of habitat quality on the supply of juveniles to adult populations. Table 1 details these assumptions, provides information on their application in the case study example, and suggests possible improvements where data are available. For example, based on reported habitat associations from the literature and observation of areas with high densities of juveniles, adult *B*. *muricatum* habitat in Yap was assumed to be the barrier reef outer slope [49] and nursery habitats were assumed to be mangroves and enclosed lagoons within the reef flat [26,45,49]. These assumptions could be refined through comparative surveys to identify the size distribution of *B*. *muricatum* in different habitats, stable isotope analysis to associate nursery habitats with adult populations, or empirical measurement of fish movement patterns (assumption 1, Table 1).

**Table 1:** Assumptions required to derive a seascape connectivity metric to inform conservation prioritisation.

Assumption	Description	Case study application	Possible improvements
1. Nature of nursery and adult habitat	Habitat patches identified as nursery and adult habitat form the basis of seascape connectivity analysis. Various definitions of nursery habitat are reviewed in [25]. Most simply, nursery habitats are considered as those contributing a higher than average biomass of juveniles per unit area to the adult population [19]. Some species may perform multiple ontogenetic shifts, and where these can be identified, the sequence of habitats used throughout ontogeny should be accounted for [25].	Habitat maps were sourced from the Millennium Coral Reef Mapping Project (http://imars.usf.edu/MC/). Based on the literature, and limited observations within Yap, adult habitat was assumed to be the barrier reef outer slope [49]. Nursery habitats were assumed to be mangroves and enclosed lagoons within the reef flat [26,49]. Houk *et al*. [45] found greater densities of juvenile *Cheilinus undulatus* on lagoon reefs in Yap, pointing to their potential importance as juvenile habitat. Though an important nursery habitat for many species [53], seagrass was not considered as a critical nursery habitat during analysis, due to its wide availability throughout the seascape. In the absence of data to suggest otherwise, lagoon reefs and mangrove habitats were assumed to have equal importance as nursery habitat.	A range of approaches can be used to identify nursery habitat(s). In order of increasing strength of inference for examining seascape connectivity: - reported habitat associations from the literature; - areas observed through visual surveys to have high densities of juveniles; - spatial and temporal patterns in the size distribution of species determined through comparative sampling in different habitats [20]; - stable isotope analysis to associate nursery habitats with adult populations [54]; - empirical measurement of fish movement patterns (see below). Where multiple nursery habitats are utilised by focal species and some are known to have greater importance than others (either in terms of functional dependency or relative contribution to adult populations), this could be accounted for by weighting the terms summed during calculation of seascape connectivity cost.
2. Maximum distance fish move between juvenile and adult habitats	The spatial extent of species’ movement capabilities informs which habitat patches should be considered to be connected. Empirical data available for some species indicate interspecific variability in the spatial scale of ontogenetic migrations: whilst some snappers (e.g. *Lutjanus apodus*) perform ontogenetic shifts of 10s–100s of metres, some jacks (e.g. *Caranx ignobilis and C*. *sexfasciatus*) move several kilometres between juvenile and adult habitats [35]. Blackspot snapper (*L*. *ehrenbergii*) have been recorded moving >30 km from coastal nursery habitats to reefs [54].	No empirical data on the distance of ontogenetic migrations in the focal species was available. It was thus assumed that individuals can migrate to adult habitat within 7.6 km of juvenile habitat, based on the maximum recorded home range size for *B*. *muricatum* [35]. Martin *et al*. [27] suggest that connectivity effects may be amplified on reefs located closest to juvenile habitat; thus, a negative exponential was used to represent preferential settlement on proximate adult habitat.	Tools and techniques for measuring fish movement (e.g. tag-mark-recapture, passive and active acoustic telemetry) have been described and discussed elsewhere (e.g. supplementary information in [35]). Where data on ontogenetic migrations is lacking, other empirical data, for example of home ranges or spawning migrations, might indicate species movement ability. However, ontogenetic migrations can cover greater or lesser distances than typical diurnal movements [35]. Maximum movement distances might also be inferred from regional-scale correlations between species’ abundance and habitat dispersion; for example Mumby [24] assumed a maximum migratory distance of 10 km, based on the maximum distance observed between offshore mangrove cays and reefs in the Caribbean.
3. Migratory pathways between nursery and adult habitat	Migratory pathways between habitats may affect the relevant distance between patches. Patterns of movement between habitats may be spatially and temporally predictable [25,55]. For example, specific routes may be preferred if they span the shortest distance between habitats, lower predation risk, or facilitate tidally enhanced movements [25]. Migratory pathways might also be influenced by local oceanography and exposure [9].	It was assumed that migrating fishes move directly between nursery and adult habitats, and are able to traverse all intermediary habitats with the exception of land and deep water.	Tracking an adequate number of individuals over the time periods and spatial extents required to establish migratory pathways is likely to be logistically and economically prohibitive in many contexts (though empirical studies might be facilitated if ontogenetic shifts are known to occur seasonally [20]). Where structure-rich corridors that lower predation risk or tidal channels that facilitate movement across the seascape can be identified from benthic habitat maps, these can be accounted for in a cost surface used to determine least cost pathways between habitats.
4. Homogenous quality of nursery and adult habitat	Aside from their location relative to adult habitats, the quality of nursery habitats is determined by their ability to support greater than average density, survival, and growth of juveniles [19]. Nursery habitat quality is likely to influence settlement rates and survivorship, and thus the relative contribution of juveniles to the adult population [53,56–58]. Whether a relationship exists between nursery habitat patch size and quality is unclear. Factors influencing selection of adult habitat by migrating juveniles are poorly understood, and proximity effects may be moderated by other aspects of habitat quality (e.g. availability of food or refuge) or density dependence [20].	Habitat quality were not available at relevant extent and resolution; therefore, habitat quality was determined by proximity to nursery/adult habitat alone.	Where information on heterogeneous habitat quality (e.g. live coral cover, structural complexity, water quality, tidal regime or salinity [19,53]) is available, this could be considered by excluding low quality habitat patches from prioritisation, adding a penalty cost to planning units containing low quality habitat to disfavour their selection, or identifying sites with high seascape connectivity but low quality habitat as priorities for restoration activities [20,24]. If information on juvenile home range size is available for focal species, this could be used to inform a minimum threshold patch size for nursery habitat.

**Fig 2:**
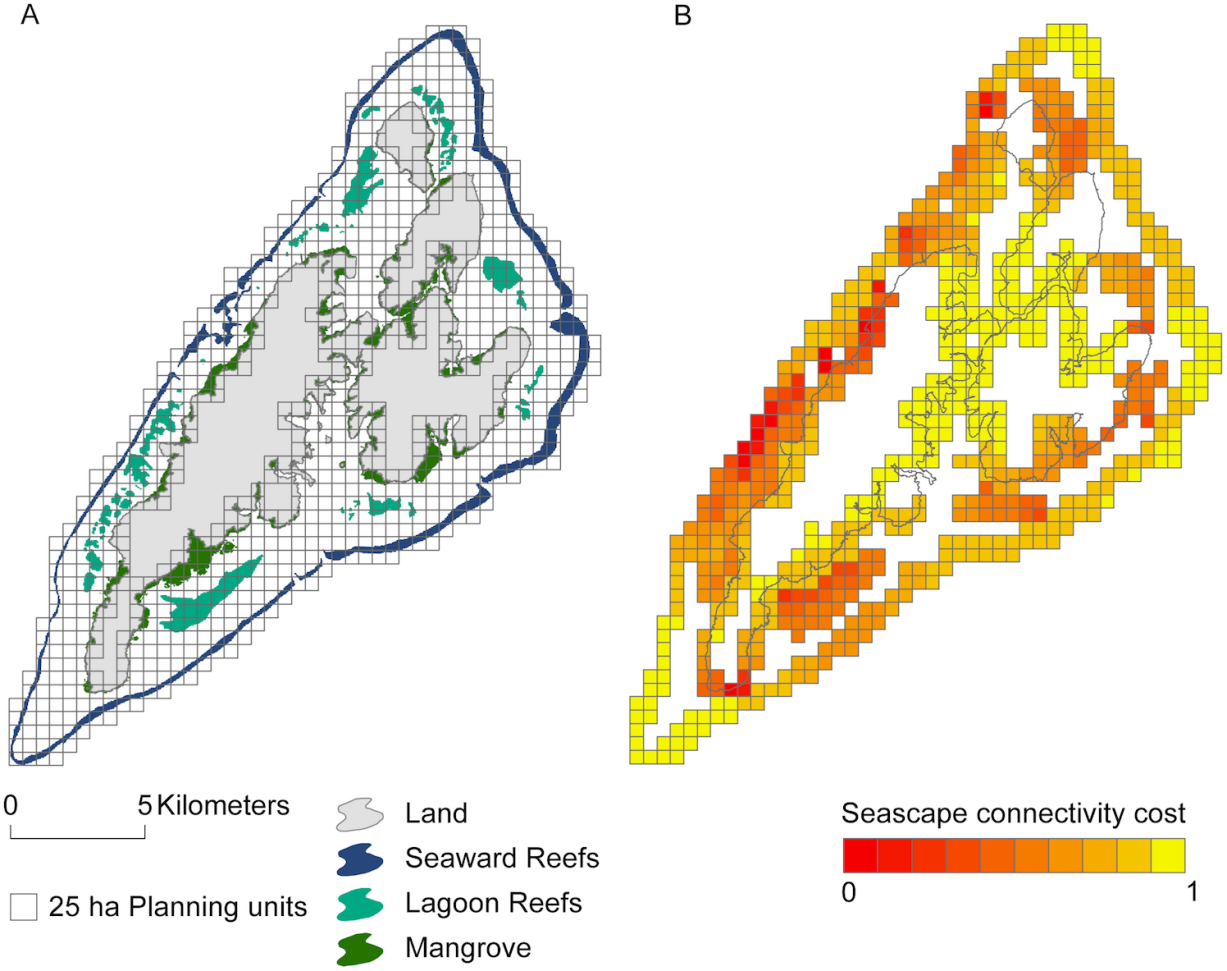
(A) distribution of mangroves, lagoon reefs and seaward reefs around Yap Proper; (B) overall seascape connectivity cost (SCC) assigned to planning units. Well-connected habitats have a reduced cost, and are thus preferentially selected by the Marxan algorithm.

To derive the seascape connectivity metric, I first divided the planning region into 25 ha planning units. This planning unit size was selected for consistency with previous conservation prioritisations conducted in Micronesia, and was considered appropriate relative to the scale at which marine protected areas have been implemented. Planning units containing critical habitat types for the focal species (i.e. seaward barrier reefs, lagoon reefs, and mangroves; assumption 1, Table 1 and Fig 2) were identified, and pairwise distances between “origin” (i.e. containing nursery habitat) and “destination” (containing adult habitat) planning unit centroids were calculated. To calculate distances “as the fish swims”, I used the ArcGIS (ESRI, Redlands CA) origin-destination cost matrix analysis tool to calculate the least-cost paths along a 30 m x 30 m network mesh (selected as a trade-off between spatial precision and computational efficiency), accounting for deep water and land barriers (assumption 3, Table 1).

The seascape connectivity value for planning units containing adult habitat (*SCA*) was calculated as:

$${SCA}_{i}=\sum_{j\in X}\left(\frac{N_{j}}{{d_{ij}}^{2}}\right)$$

where *N*_*j*_ is the area of nursery habitat in planning unit *j*, *d*_*ij*_ is the distance between planning unit *i* and planning unit *j*, and X is the set of planning units for which *d*_*ij*_ is less than a maximum threshold of 7.6 km (assumption 2, Table 1).

The seascape connectivity value for planning units containing nursery habitats (*SCN*) was calculated as:

$${SCN}_{i}=\sum_{j\in X}\left(\frac{A_{j}}{{d_{ij}}^{2}}\right)$$

where *A*_*j*_ is the area of adult habitat in planning unit *j*.

Seascape connectivity values for adult and nursery habitat patches were subsequently rescaled from 0–1 and inverted, so that a low value of the seascape metric indicates well-connected habitat. In the case study region a small number of planning units contained both nursery and adult habitat; therefore the overall seascape connectivity cost (*SCC*) for each planning unit was calculated as the minimum of *SCA* and *SCN*.

### Application in Marxan

The combined seascape connectivity metric (SCC) was included as a cost layer during spatial prioritisation using Marxan. Including seascape connectivity as a cost layer to be minimised (subject to the achievement of representation targets for conservation features) acts to differentiate between patches of the same habitat type, preferentially selecting those that are well-connected to other critical habitats in the seascape.

Given that the seascape connectivity metric is relative, and has no meaningful quantitative ecological interpretation, it is not appropriate to identify a representation target for the amount of seascape connectivity that should be included in a protected area system [1], or a threshold level above which individual planning units would be considered adequately connected (e.g. [59]). In the case study example no social or economic cost layers (e.g. opportunity costs) were available, so no trade-offs are incurred by using Marxan’s cost function to preference selection of sites with high seascape connectivity. Where socioeconomic costs need to be explicitly considered in prioritisation, related software Marxan with Zones [60] allows users to specify and minimise multiple cost layers. An alternative approach could use the area-weighted distance between planning units containing nursery and adult habitats as a “connectivity cost”, following the approach described by Beger *et al*. [61]. However, this precludes the use of a separate boundary length modifier (BLM), a parameter that allows users to express a preference for spatially clustered solutions, which was desirable in this case study.

To determine the impact of considering seascape connectivity on spatial priorities for marine protected areas in Yap, two spatial prioritisation scenarios were compared. In the baseline “equal cost” scenario, all planning units were assigned the same cost value, equal to the mean seascape connectivity cost (SCC). In the “seascape connectivity” scenario, planning unit cost values were equal to their SCC value. In line with the Micronesia Challenge objectives [62], representation targets were to include 30% of nearshore marine habitats (including mangroves) within protected areas. Variants of both scenarios were were run with and without the BLM. All other Marxan parameters were consistent across scenarios, and in the scenarios presented here, existing protected areas were disregarded.

### Identification of critical nursery habitats to improve the effectiveness of existing marine protected areas

To identify nursery habitats that are most likely to supply juveniles to adult populations within existing marine protected areas, the process of deriving the seascape connectivity metric *SCN* was repeated, using only the adult habitat destination points that fall within the boundaries of three existing, well-managed marine protected areas: the Nimpal Channel Marine Conservation Area, Reey Marine Conservation Area, and Tamil no-take zone.

## Results

The structure of the seascape in Yap means that adult and nursery habitats are generally well-connected: only the southern tip of the seaward barrier reef is >7.6 km from nursery habitat and thus beyond the expected dispersal ability of *B*. *muricatum* (assumption 2, Table 1). As would be expected, given their derivation, the spatial pattern of seascape connectivity cost values highlights areas of the seascape where nursery and adult habitats occur in close proximity. For example, the width of the seascape is narrower on the west of Yap proper, resulting in generally lower seascape metric values for reefs on the west (Fig 2B).

### Influence of seascape connectivity cost on conservation prioritisation

**Fig 3:**
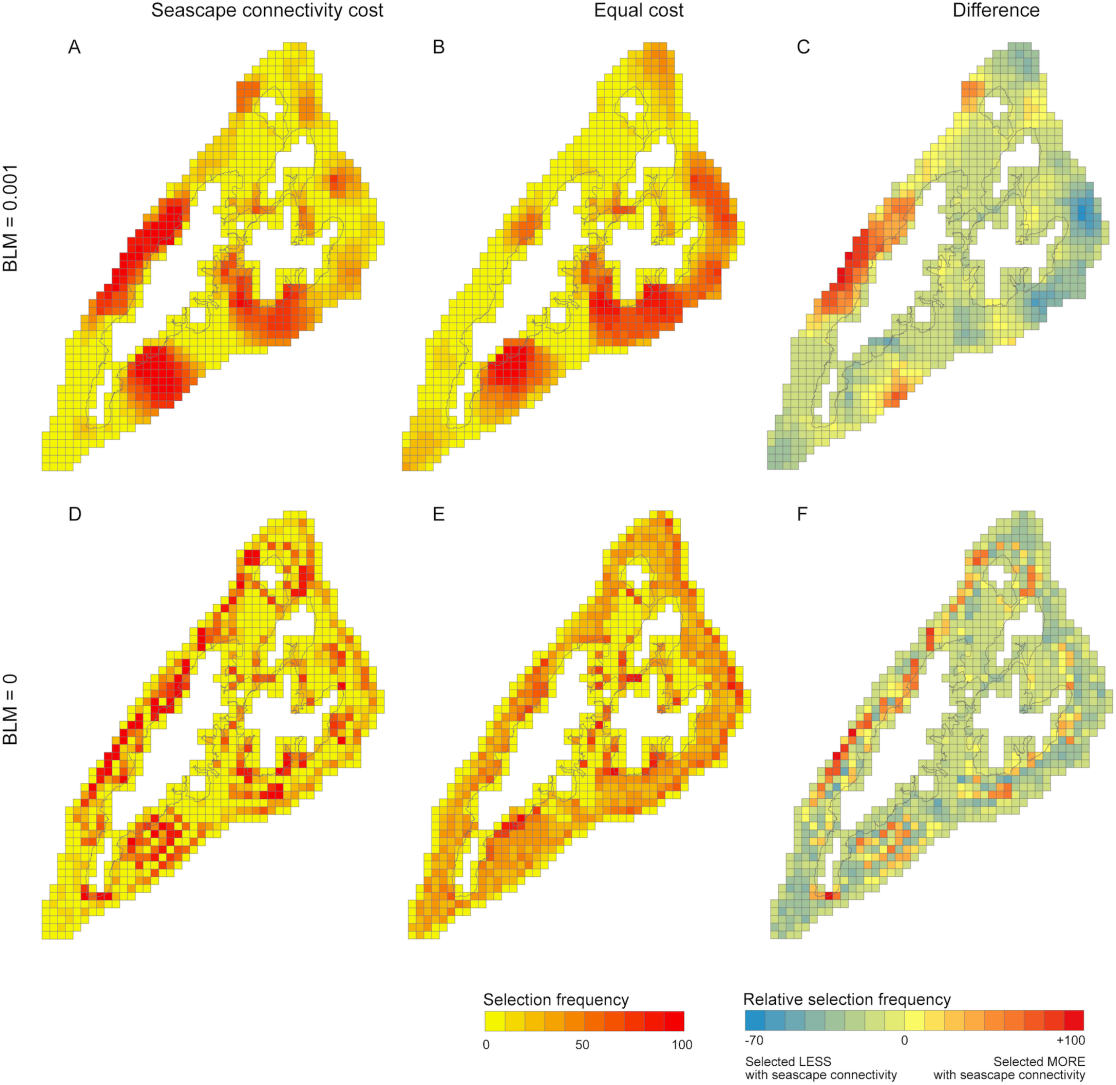
Marxan outputs comparing the selection frequency of planning units across different prioritisation scenarios: “equal cost” (A & D) and “seascape connectivity” (B & E); with (A-C) and without (D-F) the boundary length modifier. Panels C and F indicate planning units that were selected more or less frequently when seascape connectivity was considered in prioritisation.

Spatial priorities are most easily identified in scenarios where Marxan’s BLM was used to prefer spatially clustered protected area network designs (Fig 3A–3C). Planning units selected more frequently when seascape connectivity was considered in prioritisation (in red, Fig 3C) highlight three areas: reefs to the southeast of Yap proper that benefit from proximity to spatially extensive lagoon reefs and mangroves; areas on the west of Yap proper where mangroves are less extensive but all three habitat types are present and the seascape is relatively compressed; and an area in the northwest where lagoon and seaward reefs occur in close proximity. Across 100 replicate Marxan runs, planning units in these areas were selected between 60–100 times more frequently when seascape connectivity costs were used.

### Identification of critical nursery habitats to improve the effectiveness of existing marine protected areas

**Fig 4:**
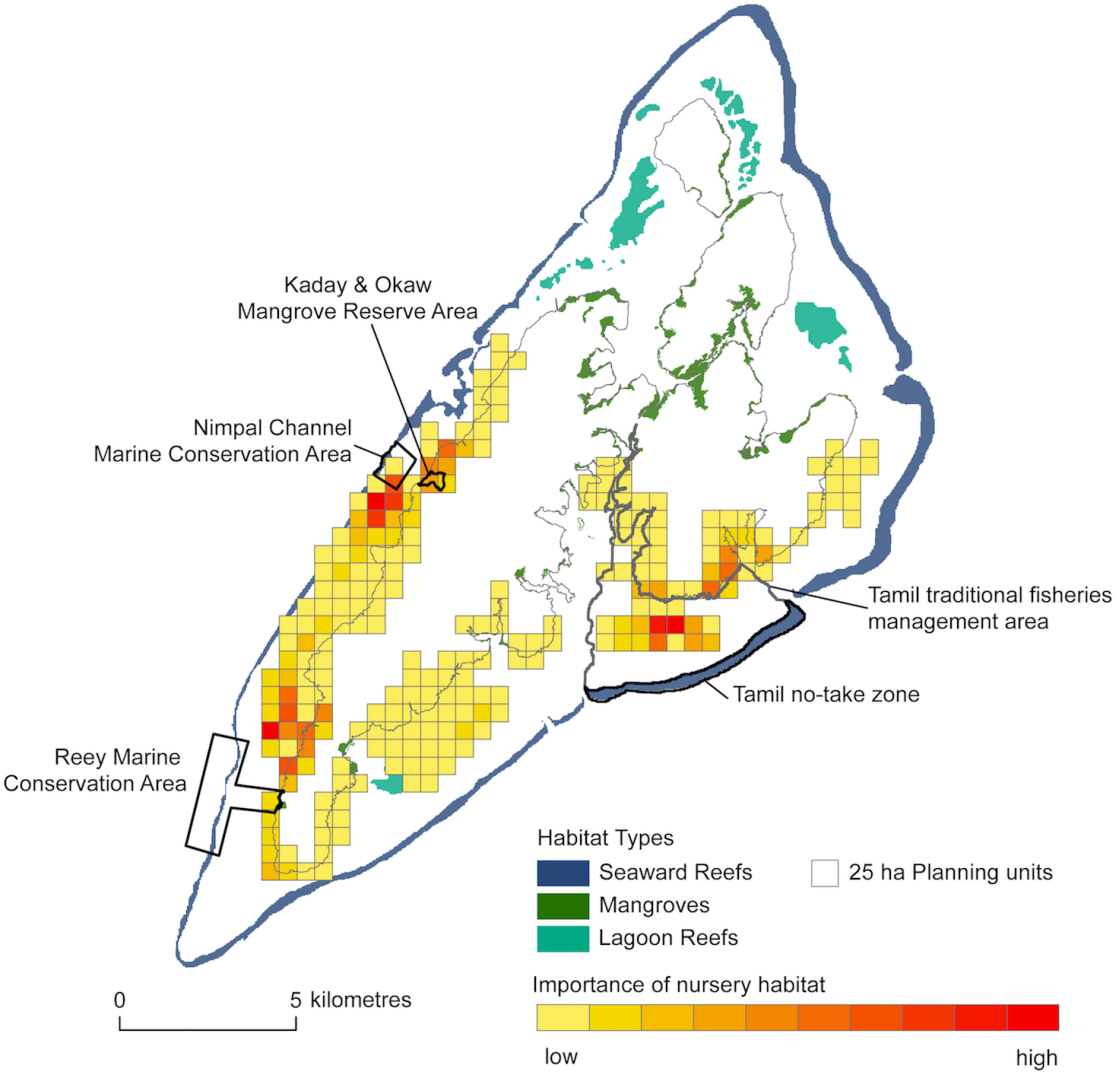
Seascape connectivity metric *SCN* indicating important nursery habitat patches where conservation might be expected to improve the effectiveness of existing marine protected areas in Tamil, Reey, and Nimpal Channel. Also indicated are the boundaries of the Tamil community traditional fisheries management area, and the Kaday & Okaw Mangrove Reserve.

Identification of habitat patches that are most likely to supply juveniles to adult populations on reefs within existing protected areas can indicate where collaboration between communities would benefit management efforts (or even be required for success). Nursery habitats most likely to supply juveniles to reefs within the Tamil no-take zone include the lagoon reefs within, and mangrove areas adjacent to, the Tamil traditional fisheries management area (Fig 4). Thus, the Tamil communities are able to implement additional management to improve the status of reef fish populations within their existing no-take zone. In contrast, the nursery habitats most likely to supply juveniles to the Reey and Nimpal Channel marine conservation areas are outside of those communities’ jurisdictions (*pers*. *comm*., community boundaries are not formally mapped, so cannot be depicted here). Fig 4 indicates that the Kaday & Okaw mangrove reserve area is likely to benefit adult fish populations in the Nimpal Channel MCA, though increasing the size of this area, and/or establishing additional management on the lagoon reefs to the south of Nimpal Channel would provide additional benefits. Similarly, the Reey community would need to coordinate with adjacent communities to ensure that nearby lagoon reefs and mangroves are appropriately managed.

## Discussion

Within the Yapese context of community tenure over resources, it is unlikely that an optimally-connected network of marine protected areas can be designed and implemented. Nevertheless, systematic conservation planning processes can help to develop a shared vision and objectives for management among stakeholders with ownership of, or responsibility for managing natural resources. Incorporating seascape connectivity in spatial prioritisation highlights areas where small marine protected areas placed on coral reefs might benefit from proximity to other habitats in the seascape and thus be more effective.

Outputs from scenarios with different BLM values can be used to guide decision-making in different contexts. For example, broader-scale priorities identified from scenarios where the BLM>0 (e.g. Fig 3A and 3C) might indicate which communities should be engaged in discussions regarding establishing protected areas; individual planning units prioritised in scenarios where the BLM = 0 (e.g. Fig 3D and 3F) could subsequently be used in those discussions to decide upon the boundaries of individual protected areas. Fig 3F shows that the seascape connectivity value of adjacent “blue holes” can vary, depending on their position relative to other habitats.

Due to the configuration of habitat mosaics in Yap, the identity of source nursery habitats for existing marine protected areas is fairly intuitive. However, this might not be the case in other seascapes. Outputs from this analysis are likely to be most useful in regions where management is highly decentralised, as is the case for many tropical developing countries with coral reefs [63].

Establishing protected areas that extend from fringing mangroves to the reef slope should remain a priority, as this will maximise protection for all species that move between habitats. Ideally, individual marine protected areas should be sized to account for species home ranges [35,64]. However, the large home range size of adult bumphead parrotfish (up to 7.6 km; [35]) means that adults are unlikely to be adequately protected within coral reef marine protected areas that can be feasibly implemented within Yap. Thus, alternative management strategies, such as increasing protection for nursery habitats (juvenile home ranges are typically smaller than those of wide-ranging adults [35]) and a ban on night time spearfishing (to which parrotfish are especially vulnerable) [49] may be most effective for improving the status of bumphead parrotfish populations.

The aim in including seascape connectivity in prioritisation was to identify habitat patches that might otherwise be overlooked for conservation. Even if a habitat patch is small in area, it can act as an important nursery habitat if it produces relatively more adult recruits per unit of area than other patches; though larger contiguous habitat patches might support a greater number of juveniles, if these individuals never reach adult populations, the value of the habitat as a nursery is reduced [19]. For this reason, I did not weight the seascape connectivity metric by the area of critical habitat within each planning unit. As a result, the application of a seascape connectivity cost emphasised habitat patches with high seascape connectivity, regardless of their area. This is perhaps most apparent for the mangrove areas on the southern tip of Yap (Fig 3F). These mangroves are not spatially extensive, but are in close proximity to the barrier reef, and thus might be important for species that perform ontogenetic migrations between mangroves and reefs, or otherwise benefit from proximity to mangroves. This result highlights the importance of validating the assumptions made in Table 1, and groundtruthing priorities prior to taking action. For example, if these mangroves are not of sufficient spatial extent or quality to support significant juvenile populations, their conservation might be unwarranted (alternatively, the area might be prioritised for restoration [24]). I made the assumption of homogenous quality of nursery habitat patches (assumption 4, Table 1). However, aside from their location relative to adult habitats, the quality of nursery habitats might vary depending on their level of larval supply, structural complexity, predation, competition, food availability, or tidal regime [19,53].

In contrast to the results presented here, weighting seascape metrics by area resulted in little difference in selection frequencies between scenarios with equal versus seascape connectivity costs. The patchy nature of nursery habitats (*c*.*f*. continuous barrier reefs) meant that the effect of patch size overwhelmed the influence of connectedness to adult habitat; i.e. larger contiguous patches of lagoon reefs and mangroves were prioritised over smaller, better-connected patches. Representation targets for individual habitats similarly preference larger contiguous habitat patches, an effect further augmented by the use of Marxan’s BLM, which acts to prioritise areas in which contiguous patches of different habitat types occur in close proximity. Thus, where nursery and adult habitats are well-connected within the extent of species’ movement capabilities and if the area of contiguous nursery habitat is considered to be important, representation targets for individual habitats types combined with a preference for spatially clustered protected area network designs could result in similar spatial priorities to those which explicitly target seascape connectivity.

Application of the seascape connectivity cost prioritised protection of coral reefs where they occur closest to nursery habitats, which in Yap are also those closest to land (Fig 3). Accordingly, Martin *et al*. [27] suggest that seascape connectivity might be incorporated in marine protected area design by simply prioritising areas where juvenile and adult habitats are closest. However, proximity to human populations correlates with both land-based pollution and fishing pressure, which negatively impact on juvenile habitats and adult populations, respectively [45,62]. Whilst anticipated threats to coastal nursery habitats underpin the importance of a seascape ecology approach, where this equates to prioritising reefs closer to land, increased opportunity costs are likely to be incurred as a result of greater fishing pressure on more accessible reefs. The magnitude of this trade-off will depend upon both the structure of the seascape and spatial patterns of fishing effort. Where opportunity costs can be borne or offset, fish populations on reefs close to human populations and historically subject to fishing pressure might be expected to benefit most from protection [65], especially where proximate, high quality nursery habitats can also be protected.

In conservation prioritisation it is commonly implicit that representation targets for habitat types are surrogates for the species which inhabit them [66]. Yet for some species, combinations of different habitat types may be more important than any single habitat [20]. This suggests that approaches to prioritisation that do not consider spatial relationships and /or connectedness between habitats may not be adequate for those species. Emerging theoretical evidence suggests that in many contexts, seascape connectivity might be more important that larval dispersal in determining the effectiveness of marine protected areas [56]. Though ontogenetic migration was not modelled explicitly, Cabral *et al*. [56] considered a three-stage population model comprised of larval, juvenile, and adult reef fish populations, with juvenile settlement and recruitment to the adult population limited by the carrying capacity of juvenile and adult habitats, respectively. They found that for a range of different larval connectivity structures, prioritising sites for protection on the basis of habitat extent and quality, rather than larval connectivity metrics, maximised metapopulation abundance. This is likely to be especially pertinent when planning at relatively small spatial scales, where it is probable that all or most habitat patches will be well-connected via larval dispersal [67]. Approaches to conservation prioritisation that consider the functionality provided by mosaics of different habitats are thus warranted.

Where primary research is impracticable, approaches that make best use of available data can be valuable [68]. Habitat maps commonly form the basis for conservation prioritisation, and it is therefore relatively straightforward to incorporate spatial pattern metrics (e.g. habitat isolation, area and proximity). Whilst care must be taken to ensure that connectivity metrics are scaled to focal species of interest and interpreted appropriately, the approach demonstrated here shows the feasibility of moving beyond generic rules of thumb for seascape connectivity even in relatively data-limited contexts. In contrast, predicting patterns of larval dispersal and considering these in prioritisation may be prohibitively difficult in regions lacking high resolution hydrodynamic data [12].
